# Community-Based Dialysis in Saskatchewan First Nations: A Grassroots Approach to Gaining Insight and Perspective From First Nations Patients With Chronic Kidney Disease

**DOI:** 10.1177/2054358120914689

**Published:** 2020-05-20

**Authors:** Lindsay Richels, Wilson Sutherland, Kyle Prettyshield, Joanne Kappel, Michelle McCarron, Bonnie Richardson

**Affiliations:** 1 College of Medicine, University of Saskatchewan, Regina, Canada; 2 Federation of Sovereign Indigenous Nations, Saskatoon, SK, Canada; 3 Saskatchewan Health Authority, Regina, SK, Canada

**Keywords:** First Nations, CKD (chronic kidney disease), home dialysis, hemodialysis, peritoneal dialysis, qualitative

## Abstract

**Background:**

Renal replacement options or dialysis can be delivered in the home setting or hospital setting. Home dialysis offers a number of benefits over hospital-delivered dialysis. These advantages include improved quality of life, less travel, and fewer dietary restrictions. Despite the benefits, home-based dialysis therapies are significantly underutilized by First Nations with only 16.2% uptake versus 25.7% uptake in non-First Nations people in Saskatchewan. It is important to recognize that First Nations have a greater burden of end-stage renal disease including higher prevalence, younger age at diagnosis, increased severity of disease, mortality at an earlier age, and increased travel distance to access kidney services.

**Objective:**

The goal of this study is to identify the existing barriers to home peritoneal dialysis and provide insight for future programs in Saskatchewan First Nations communities in a culturally meaningful framework.

**Design:**

Through qualitative research utilizing sharing circles and individual interviews, barriers to utilizing home-based dialysis were identified.

**Setting:**

Four sharing circles were held and interviews were conducted with four First Nations dialysis patients.

**Participants:**

Total number of participants in sharing circles were 67. Sharing circles were composed of patients with chronic kidney disease, patients on hospital-based dialysis, patients on home-based peritoneal dialysis, family members, health care providers (nurses, physicians, dietitians, primary care director, and coordinators). Face-to-face interviews were conducted with four First Nations dialysis patients.

**Measurements:**

The data from the sharing circles and interviews were transcribed and analyzed by a PhD researcher using constructivist grounded theory, with elements of narrative inquiry to ascertain participants’ experiences of care. Data were coded and then grouped into categories using qualitative research software NVivo. Saturation of data was achieved.

**Methods:**

Documenting and recounting patient and community experience with chronic kidney disease through sharing circles involving patients, family members, and health care providers has been the central information base for this project. Qualitative interviews were conducted with patients who currently use home dialysis and those who travel to hospital for dialysis. Written consent was obtained from all participants. Information was gathered via audio recording of all sharing circles and interviews. Transcription of the interviews was completed with confidentiality maintained during transcription.

**Results:**

The main theme of our results was addressing the underutilization of home-based peritoneal dialysis in First Nations Communities. Five subthemes emerged from the main theme and included logistics, education and information, training and support, community support, and culture and leadership. Through sharing circles, a secondary theme of observations about living with chronic kidney disease and experiences of being on dialysis was explored.

**Limitations:**

A small number of First Nations communities were involved in this project, and although the data reached saturation, we cannot presume that the information is representative of all First Nations in Saskatchewan. There were a limited number of patients currently on home-based peritoneal dialysis, and therefore their perceptions may not be adequately captured. Participant characteristics (patient, caregiver, nurse, etc) were not captured when speaking in the sharing circles, and therefore participants are not classified when quoted.

**Conclusions:**

Strategies to help improve home-based dialysis included improved education, local support, integrated traditional medicine, cultural sensitivity, and leadership prioritization.

## Abrégé

**Contexte:**

La dialyse et les autres modalités de remplacement rénal sont offertes en centre ou au domicile du patient. La dialyse à domicile offre de nombreux avantages comparativement à la dialyse en centre, notamment une meilleure qualité de vie, et requiert moins de déplacements et de restrictions alimentaires. Néanmoins, cette modalité continue d’être sous-utilisée par les patients autochtones en Saskatchewan (16,2 % contre 25,7 % dans la population allochtone). Il convient de souligner que le fardeau de l’insuffisance rénale terminale est plus important chez les patients autochtones: prévalence plus élevée, diagnostic et mortalité plus précoces, sévérité accrue de la maladie et distances plus grandes à parcourir pour accéder aux services néphrologiques.

**Objectifs:**

L’étude vise à recenser les facteurs limitant l’adoption de la dialyse péritonéale à domicile comme modalité, et à fournir un aperçu des programmes à venir dans les communautés des Premières Nations de la Saskatchewan dans un cadre culturellement significatif.

**Type d’étude:**

Nous avons recensé les obstacles au choix de la dialyse à domicile comme modalité de traitement par l’entremise d’une recherche qualitative fondée sur des cercles de partage et des entretiens individuels.

**Cadre:**

Quatre cercles de partage ont été tenus et des entretiens ont été conduits auprès de quatre patients autochtones dialysés.

**Participants:**

Au total, 67 personnes ont participé aux cercles de partage composés de patients atteints d’insuffisance rénale chronique, de patients dialysés en centre, de patients dialysés à domicile, de membres de leurs familles et de fournisseurs de soins (infirmières, médecins, diététistes, coordinateurs, administrateurs de centres de soins de santé primaires). Quatre patients autochtones traités en dialyse ont été questionnés individuellement.

**Mesures:**

Pour catégoriser les expériences de soins, les données recueillies au cours des entretiens et des cercles de partage ont été transcrites et analysées par un chercheur doctorant à l’aide d’une théorie constructiviste fondée sur des éléments d’enquête narrative. Les données ont été codées et groupées en catégories grâce au logiciel d’analyse qualitative NVivo. La saturation des données a été atteinte.

**Méthodologie:**

La base d’information centrale de ce projet a été de documenter et de relater l’expérience des communautés autochtones et des patients atteints de néphropathie par l’entremise de cercles de partages impliquant leurs familles et des fournisseurs de soins. Les entretiens qualitatifs ont été menés auprès de patients pratiquant la dialyse à domicile et de patients recevant leurs traitements en centre hospitalier. Le consentement écrit a été obtenu de tous les participants. L’information a été recueillie à partir de l’enregistrement audio des entretiens et des cercles de partage. La transcription des entretiens a été complétée dans le respect de la confidentialité.

**Résultats:**

Le thème principal de notre recherche était d’aborder la sous-utilisation de la dialyse péritonéale à domicile comme modalité dans les communautés autochtones. Cinq sous-thèmes ont découlé du thème principal, soit: a) la logistique, b) l’éducation et l’information, c) la formation et le soutien, d) le soutien de la communauté, et e) la culture et le leadership. Un thème secondaire a été exploré par l’entremise des cercles de partages, soit la collecte d’observations concernant la vie avec l’insuffisance rénale chronique et l’expérience d’être traité en dialyse.

**Limites:**

Peu de communautés autochtones ont été impliquées dans ce projet et bien que les données aient atteint la saturation, nous ne pouvons présumer que l’information recueillie est représentative de tous les membres des Premières Nations de la Saskatchewan. Un faible nombre de patients était traité par dialyse à domicile; dès lors, leurs perceptions pourraient ne pas être saisies adéquatement. Les caractéristiques des participants (patients, fournisseurs de soins, infirmières, etc.) n’ont pas été colligées lors des cercles de partage et ainsi, les participants ne sont pas classés lorsque cités.

**Conclusion:**

Les stratégies visant à favoriser la dialyse à domicile incluaient l’amélioration de l’éducation, le soutien local, l’intégration des pratiques de la médecine traditionnelle, une approche tenant compte des différences culturelles et la priorisation de la part des intervenants.

## What was known before

First Nations patients have significantly more burden with chronic kidney disease yet their utilization of home peritoneal dialysis therapies are significantly lower.

## What this adds

Through sharing circles and interviews, participants identified delivery of information, lack of community-led training and support, low prioritization by leadership, cultural differences between patients and their care providers, inability to self-manage treatments because of physical or environmental limitations, and logistical shortcomings with housing, hygiene, storage, and supply as barriers to home-based peritoneal dialysis.

## Introduction

Renal replacement options or dialysis can be delivered in the home setting or hospital setting. Home peritoneal dialysis offers a number of benefits over hospital-based dialysis. Home peritoneal dialysis offers more uninterrupted time for work, family, and social activities, but it does require training and dexterity to connect and disconnect the machine.^1^ One of the drawbacks to home peritoneal-based dialysis is an increased risk of catheter site infection and peritonitis when compared with hospital-based dialysis.^1^ Hospital-based dialysis does not require training but it does require travel and time away from home which means many patients are no longer able to work because of the time requirements for dialysis. During hospital-based treatments, because of the rapid changes in fluid balance many patients get hypotensive and can become symptomatic with lightheadedness, shortness of breath, abdominal cramps, nausea, or vomiting.^1^ Overall home peritoneal dialysis has many advantages over hospital-based dialysis, including improved quality of life,^2^ less travel,^2^ and fewer dietary restrictions.^3^ An environmental scan with First Nations in Saskatchewan in the 2015 *Epidemiological Scan of the Burden of Chronic Kidney Disease in Saskatchewan’s First Nations People*^4^ demonstrated that First Nations patients were significantly more burdened with chronic kidney disease than non-First Nations. This research showed that First Nations have a much higher prevalence of kidney disease, are younger at diagnosis,^5^ have more severe kidney disease, die younger, and travel further to receive kidney services when compared with non-First Nations. Although First Nations patients had significantly more burden with chronic kidney disease, their utilization of home dialysis therapies are significantly lower. First Nations utilization of home dialysis was only 16.2% versus 25.7% uptake in non-First Nations people.^6^ Previous qualitative research indicates First Nations people with end-stage kidney disease have an overwhelming source of burden, frustration, and economic hardship when placed on hospital-based dialysis.^7^

Home peritoneal dialysis is significantly more cost effective for the health care system than hospital-based dialysis. In Canada, the total annual health-care cost of treating a patient with end-stage renal disease (ESRD) using in hospital dialysis is almost doubled annually ($95 000-$107 000) when compared with home peritoneal dialysis ($56 000),^8^ marking a substantial savings when home peritoneal dialysis is implemented. This annual figure does not include the tangential costs to family members taking time off work; time spent driving, or purchasing meals away from home.

Home peritoneal dialysis offers better quality of life, lessens the burden of travel, requires fewer dietary restrictions and is approximately half the cost of hospital-based dialysis yet is significantly underutilized by First Nations communities. The reasons for the disparity in utilization are unknown and the barriers to home dialysis in First Nations communities in Saskatchewan remain unclear. The goal of this study is to identify the existing barriers to home peritoneal dialysis and provide insight for future programs in Saskatchewan First Nations communities in a culturally meaningful framework.

## Methods

### Study Design

The Saskatchewan Kidney Program, Federation of Sovereign Indigenous Nations (FSIN), Meadow Lake Tribal Council (MLTC), Touchwood Agency Tribal Council (TATC), and All Nations Healing Hospital (ANHH) initiated this research project with the goal of improving access to home-based dialysis for First Nations people. The project utilized qualitative research to gain better understanding of the barriers First Nation’s peoples face in implementing home dialysis treatments and gain insight into opportunities for improving home dialysis uptake among First Nation’s people. First Nations peoples historically have utilized sharing circles as a healing method in which all participants (including the facilitator) are viewed as equal and information, spirituality, and emotionality are shared.^9^ In a research setting, sharing circles are concerned with gaining knowledge through discussion; the principles behind a sharing circle are quite different from other qualitative research methodologies in that circles are acts of sharing all aspects of the individual—heart, mind, body, and spirit and permission is given to the facilitator to report on the discussions.^10^ It is important to keep in mind that conducting research with First Nations utilizing externally driven quantitative survey-type methodology is considered unacceptable.^11^ There are several formats for conducting circles which can be adapted based on the desires of the group and through consensus with the group on how to proceed.^9,10^ Aboriginal spiritual ceremonies and sacred objects, the use of “rounds,” a talking object or protocol for speaking turns, and respect for all voices as personal truths are common elements of sharing circles.^9,10^ Documenting and recounting patient and community experience with chronic kidney disease through sharing circles involving patients, family members, and health care providers has been the central information base for this project. Four community sharing circles were held, 3 in MLTC and 1 in TATC.

Qualitative interviews were conducted with patients and families, both those who currently use home dialysis and those who travel to hospital for dialysis, in order to gain a better perspective on the challenges, barriers, and benefits of home dialysis for First Nations. Interview data were analyzed using constructivist grounded theory,^12^ with elements of narrative inquiry,^13,14^ in order to ascertain participants’ experiences of care. Four interviews were conducted with ANHH patients.

Harmonized ethics review was undertaken by the Ethics Review Boards of the University of Saskatchewan and Regina Qu’Appelle Health Region. The project received ethics approval on September 14, 2016 (REB-16-84). The project received funding under the Saskatchewan Health Research Foundation’s Collaborative Innovation Devel-opment Grants Program in 2016. Ownership, Control, Access, and Possession (OCAP)^15^ principles for research with First Nations communities, as well as the importance of community engagement as described in Chapter 9 of the second edition of the Tri-Council Policy Statement (TCPS 2), were respected and followed throughout this study.

#### Sharing circles

Sharing circle gatherings of kidney patients, family members, and health care providers in MLTC and TATC were planned and designed to learn about patient and provider experiences with kidney disease services and views about home-based dialysis options, and to explore patient/provider ideas about how services should be designed. In summary, the project sought participant advice with respect to how home-based options should be designed to meet patients’ needs.

Written consent was obtained from all participants. Information was gathered via audio recording of all sharing circles and interviews. Transcription of the interviews was completed with confidentiality maintained during transcription.

Each sharing circle was designed to include 10 to 15 patients and service providers representative of MLTC and TATC communities. Participants were recruited via invitation sent by the community health director. Invitations were sent to patients, family members, and providers in surrounding communities. Participants were provided an honorarium for their participation. Informed consent was obtained at the outset of each meeting. Consent forms were approved by a harmonized ethics review undertaken by the University of Saskatchewan and RQHR Ethics Review Boards. All sharing circles were audio recorded with the consent of participants.

Three sharing circles were held in MLTC:

1. *Ministikwan First Nation, December 7, 2016.* In attendance 16 individuals: 11 patients (3 dialysis patients), one health center staff person, MLTC nurse, MLTC nutritionist, MLTC Primary Care Director, and the project coordinator.
2. *Waterhen Lake First Nation, February 9, 2017.* In attendance 16 individuals: 1 patient, 10 family members, 2 local health care providers, nephrologist, MLTC Primary Care Director, and the project coordinator.
3. *Canoe Lake First Nation, February 14, 2017.* In attendance 13 individuals: 2 patients, 4 family members, 3 local health care providers, nephrologist, MLTC nutritionist, MLTC Primary Care Director, and the project coordinator.

One sharing circle was held in TATC:

4. *Muskowekwan First Nation, January 31, 2017.* (Note this was a Tribal Council event and 3 First Nations were represented—Kawacatoose, Gordons, and Muskowekwan.) In attendance 22 individuals: 10 kidney patients, 5 family members, 3 local health care providers, 2 nephrologists, RQHR home dialysis coordinator, and the project coordinator.

#### Interviews

A total of 4 First Nation patients were interviewed as part of the project. Of the 4 patients interviewed, 3 utilized hospital-based dialysis and 1 utilized home-based dialysis. Interviewees were approached by community health directors and asked whether they wished to participate in an interview about their experiences with kidney disease services, views about home-based dialysis options and to explore patient/provider ideas about how services should be designed. If patients were open to participating, their contact information was passed on to the interviewer. Interviewees were contacted by phone to set up initial meetings. During the initial meetings, the project was described and consent forms were presented and discussed. Informed consent was obtained before the interview process commenced. Consent processes and forms were approved by the harmonized ethics review undertaken by the University of Saskatchewan and RQHR Ethics Review Boards. Three individuals were interviewed during dialysis treatment at the Regina General Hospital Renal Unit. One individual was interviewed in her home on a Saskatchewan First Nation. Once transcribed the content of each interview was reviewed and vetted with the interviewee. All interviews were audio recorded with the consent of the patient. Participants were provided an honorarium for their participation.

## Data Analysis

The data from the sharing circles and interviews were transcribed and analyzed by a PhD researcher using constructivist grounded theory,^12^ with elements of narrative inquiry to ascertain participants’ experiences of care. Data were coded and then grouped into categories using NVivo software. Saturation was achieved.

## Results

The main theme of underutilization of home dialysis was central to the data extracted from the circles and interviews. Identified within the main theme of underutilization of home-based dialysis bore 5 subthemes as participants reflected on chronic kidney disease and dialysis experiences. The 5 subthemes were logistics, education and information, training and support, community support, and leadership and culture. A secondary theme of living with chronic kidney disease emerged as people wanted to share their story. The topics discussed were not necessarily related to the type of dialysis they chose but rather sharing their story of living with chronic kidney disease and the factors that contributed to requiring dialysis in the first place. The secondary topics included in the theme “living with chronic kidney disease” were caregiver burden, the physical and mental health effects of being on dialysis, the burden of traveling, social determinants of health, and broader health care needs of the community.

### Logistics

This subtheme revealed the many challenges to implementing home-based peritoneal dialysis in rural and remote locations and the necessary infrastructure and services needed to make home-based peritoneal dialysis a realistic option. There were 4 main types of logistical issues: equipment, infrastructure, supplies, and services.

#### Challenges

Equipment is costly and if it malfunctions it needs to be addressed in a timely manner. Having the necessary infrastructure in place can be a large barrier for the communities engaged in this project as water, electricity, and storage space can all have potential issues. A few key pieces including the supply of potable water was discussed. For some communities, there would be challenges obtaining enough storage space to contain clean water. Reliable electricity was identified as a barrier in some communities they can go days without electricity.

Nurse Practitioner: She used to do her dialysis there. She did very well. But, I mean, she was an exception. And there wasn’t very many of them then. Like, we have a lot more diabetics now, we have a lot more people with chronic disease management who have renal issues. So, there’s almost not enough machines to go around, and as we get more, like if we had 56 diabetics here now, and even four of those become, have kidney failure, that’s four more people who need blood dialysis. At some point, there’s more people that need it and not as many machines available. (Other Participant: “What if we had a system set up here?”) It would be very unlikely we’d get a blood system set up here based on the water supply and inadequate power. Look at the power, we had three days out, that would ruin the machines. You’d have to have a back-up generator and all that sort of stuff. But there’s no reason why we couldn’t assist people doing peritoneal dialysis here. We have all the skills here.

Getting supplies to the community was one issue; the other was storage space of an entire month of supplies.

And a person that lives with a bunch of people in his family, where does he put all this stuff? Like, you have to get it every week in order to have space to store it. If you’re using a whole room for one person a month, families have more than one person in their house, so physically probably there’s not a lot of room to store that stuff . . . (This was addressing the requirement of having to store supplies for an entire month as that was the delivery schedule.)

A participant was offered home-based peritoneal dialysis and had a close friend who was a strong advocate for home options because of improved quality of life and “autonomy from the machine” but chose center-based dialysis because of concern surrounding water quality in the community and a fear of needles.

Another participant indicated they were well-informed about home-based dialysis options and had even had an incision made for peritoneal dialysis but developed complications. They also initiated the process to get home hemodialysis but never heard a response. Although they were willing to try, home-based options were challenged by obstacles and grew in frustration with the difficulty in completing the process. Also, heard was a participant who chose center-based hemodialysis as his schedule was better accommodated with frequent travel to other cities.

#### Solutions

Participants reported good experiences with working with device manufacturers to replace malfunctioning equipment in a timely manner. Other suggestions included working with local health center (eg, have a room in the facility dedicated to peritoneal dialysis where the patient can come in so that a room in their home is not being taken up, and where supplies can be stored). This solution can present its own set of challenges, though: including access to the facility and additional funding for staff. Another solution discussed was potential satellite centers in larger communities such as Loon Lake, Meadow Lake, and North Battleford. A heated, central supply warehouse with 24-hour access was suggested. That way, all home-based peritoneal dialysis patients in the community could store this in one place, which may help to address the issue of room for storage in their homes. Having a dedicated peritoneal dialysis resource person and consistent dietitian to support the program and potentially prevent others from requiring dialysis.

### Education and Information

This subtheme reflected on instances in which participants had questions about chronic kidney disease and various dialysis options. Formal clinical terms such as “peritoneal dialysis” are not necessarily meaningful to people without a background in health care. Some patients and families had misconceptions about what does and does not contribute to chronic kidney disease in the first place. Education about home-based peritoneal dialysis and its overall safety as compared with hospital-based hemodialysis may be needed to help dispel fears or hesitations based on anecdotal information.

#### Challenges

Some participants lacked knowledge about chronic kidney disease—the cause of chronic kidney disease, the progression of the disease, and the terminology used when health care professionals discussed chronic kidney disease. One person thought that stress was the cause of chronic kidney disease. The different terminology used, such as the difference between peritoneal dialysis and hemodialysis, was not well understood.

Nurse Practitioner: One of the things that was significant when they were doing the screening here was the fact that people don’t always understand what diabetes is, what dialysis is, what that means, how their lifestyle affects them. Some people said, ‘don’t test me, I don’t want to know. If I get it I’m going to die anyway.’ And that’s really not true. The thing is, some people refuse screening because they didn’t want to know because they were afraid. So there’s a big lack of knowledge about how to manage diabetes, how to manage kidney failure, what sort of things are available and what are not available.

Patients expressed hesitancy to try home-based peritoneal dialysis if they knew a person who had a bad experience and generalized this experience to peritoneal dialysis as a whole. Leading to the perception that complications such as infection were greater than the actual incidence. There is quite a learning curve when it comes to doing home-based peritoneal dialysis for the patient and the caregiver/support person which can be intimidating. Both patients and caregivers voiced the concern “what if something goes wrong.”

Some participants were informed about the option of home-based dialysis; some received minimal information or unhelpful information; and some based their decisions on anecdotal information.

[Interviewer:] So you’re aware that there are home based options called peritoneal and home hemodialysis? Has anybody ever talked to you about-? [Participant:] No, they just give me pamphlets from the hospital but I can’t read that good, so I don’t bother with them . . .

One participant had known 3 individuals on home dialysis and all had died early. Another participant indicated the information that was provided was placating instead of being realistic about the severity and trajectory of the disease.

Nobody took the time to explain what dialysis is? What kidney failure is? What renal failure is? And the seriousness of it.

One patient indicated they did not take the diagnosis of chronic kidney disease seriously because she did not understand the severity of the disease.

“Cancer is taken much more seriously because people understand the severity.”

#### Solutions

There are a variety of ways that educational information about chronic kidney disease (including its causes), dialysis in general, and home-based peritoneal dialysis in particular can be distributed. A multimodal approach, that is, using different means of communication in an effort to reach as broad an audience as possible, was recommended. Participants suggested using e-mail, online resources, printed material, local news media, newsletters, pamphlets, and social media. All information needs to be direct and clear—present a simple, straightforward message. It is also important to recognize that when educational material is provided, some people may struggle with written material. An alternative way of educating people and sharing information could be via traditional storytelling methods.

### Training and Support

For home-based peritoneal dialysis to be a realistic option, not only are the infrastructure and services (see subtheme “Logistics”) needed to support this, but there must be investment in providing one-on-one training to patients and caregivers to initiate treatment and ensure ongoing support is made available.

#### Challenges

Many participants felt unqualified, ill prepared, or uninformed to self-manage dialysis at home. One interview participant indicated his wife is worried about doing something wrong and the amount of time it would take to reach a hospital should a complication arise. Another participant, who became a proponent for peritoneal dialysis after experiencing center-based dialysis, said, “At first it was scary because it seemed like there was so much you had to learn before you could actually do the therapy at home by yourself . . . ” Another participant echoed previous comments that they felt better supported by the professionals at the dialysis unit than they would feel managing at home alone.

Two participants had never considered home-based dialysis because of blindness and no assistance in living with blindness. One participant had been blind for 2 years and had not received any training about how to live with blindness. She considered peritoneal dialysis but because she’s blind and “doesn’t want to do that to her boys” she has not pursued home-based options.

#### Solutions

Many solutions identified were acknowledged as already in place; however, participants wanted to reiterate and emphasize the importance of continued funding and utilization of the supports already in place. They also found the orientation program informative and the questions they had after training were adequately answered via telephone.

The training there was like two days and it was really awesome, it just prepared us for when we were going to begin at home, but still in the beginning in the first couple weeks or something, we’d have to, we found ourselves calling the hospital so often, and they were so helpful, they’re really helpful. They answer your questions, they guide you through, you know say if the machine is alarming because the machine does do that, it comes at you and says that something is wrong or something is set up not right so the machine won’t be able to work so the first couple weeks were the scariest but after that as we learned, like everyone would hook up the machine, like they’d set up the beds for us and it got easier and easier and easier and you know to the point where it became a routine thing and became easy.

### Community Support

In addition to the one-on-one supports from health care providers to patients and caregivers outlined in subtheme “Training and Support”, support is needed for—and by—the community as a whole. Extended family, friends, and other members of the community can be, and often are, great resources for dialysis patients and their caregivers. When patients need to travel long distances often to receive hospital-based dialysis, people from the community may end up providing rides and supporting the family in other ways. This can take its toll not just on the patient and caregiver, but those in these secondary support roles as well.

#### Challenges

Caregiver burden was identified as an ongoing issue. Some individuals spoke about the need for more consistent local support such as home care as well as local role models, champions, and mentors. People also spoke to the importance of local political leadership to support people on home dialysis.

Supports (dietitian, nutrition) are not consistent.

There is a need for one-on-one supports for people in community. Another individual had recently started on peritoneal dialysis and indicated the initial adjustment was the most difficult and increased support from homecare would be beneficial. With respect to kidney disease and dialysis resources must be allocated at the local level from screening to follow up local resources are necessary.

Who’s gonna pay? Who’s gonna think it’s important enough?

#### Solutions

One solution that was proposed to consider creating specialized group homes on reserve which would allow for storage of materials, consistent home care and follow-up, ensure water and electrical requirements are met as well as liaise with hospitals. Throughout the sharing circles, there were several positive things going on locally that were identified including band support for making changes in the home to accommodate home-based peritoneal dialysis and the care communities have for one another. One participant gave the example of a time when their spouse fell, and she could not help him up; her son called a friend who lived on a reserve close to where they were and the friend came out and helped. Participants had a strong sense of community and importance of helping one another.

Yeah, I used to drive a medical van and I used to give medical to dialysis before and I was never taught it before and I know how hard it is. I used to wait for my grandmother when I was a little girl, she gave me an Indian name [unclear] here you have to help people, that’s your Indian name. She said my granddaughter, don’t ask for money, or anything and always help people out. I kind of grew up on a reserve and there’s wakes and funerals, like we cook all night for them and help them out. Even people walking to town, I usually give them a ride like young people. I was a school bus driver and a lot of times they go to a white school and 4 or 5 dollars to help them buy a drink or pop or whatever and that’s how I kind of, my grandmother kind of raised me up like that. Yeah, so I really took a look into the people and help the people out on the reserve. We’re all Indians, that’s what my grandmother said, we’re all Indians. My granddaughter you’ll never be a white man so it kind of told me what an Indian had to be and all that.

### Leadership and Culture

To effect any real change, there need to be champions for this cause, including people who are respected within the health care system and have some power/influence to promote and drive change, as well as cultural leaders.

#### Challenges

Challenges identified included language barriers, system-based barriers, and the burden on First Nations people to access health care. With regard to paying for services and remuneration for expenses, the system is not set up with the people in mind and the reimbursement for travel costs does not offset the actual costs of traveling to receive dialysis services.

There is this jurisdictional stuff that gets in the way all the time, right? . . . . I’d toss jurisdiction out the window. You know, well, people should be served first and then worry about who pays. That’s my perspective on it.

There can be a general mistrust of the health care system. The effects of colonization are still felt and mistrust in service providers and modernization is also a concern. Some Elders in the community were very suspect of treated water and chose to drink lake water.

Our people are commodities.

People can feel oppressed or dehumanized. Discrimination can be systemic (eg, disregarding traditional healing alongside western medicine) or overt (eg, being treated differently because of race).

The systems don’t value human life. All they see is this and our people are not that to us. Our people are human beings and our children deserve to have a future without, we expect illness like colds, flu and things but not to know up ahead what’s in store for them right now, that’s where we are. The vision is bleak.

Dr. seen me fight with Health Canada right in the office. They still put these oppressive policies. We don’t have dietitians out there, you know, funding is capped.

Others recognized the importance for health care providers to understand First Nations history and cultural practice.

First Nations people are not apt to demand things while other cultures do demand things. Many First Nations cultures show respect to authority by not making eye contact. We respect people in authority and assume they are telling the truth. First Nations people on wards will sit quietly and not ask for anything. We need to begin to ask.

#### Solutions

One solution is to have translators available for those who would like support from the health care system but do not speak English (or speak it to a limited extent). Several participants emphasized the importance of Elders, traditional medicines and foods, and local leadership in supporting renal patients. Health care providers may benefit from cultural advisors to understand and respect cultural practices and recognize the importance of family ties. Having champions for the cause could be of benefit especially to get the attention of the health care system and government.

Suggestions for improving uptake of home-based dialysis includes enhanced social and emotional support in the community in the form of peer support from others on dialysis. Also, having Elders or traditional healers in the community involved in care would be beneficial. Increasing awareness about dialysis and chronic kidney disease in the community is important so people are better able to support one another.

### Secondary Theme: Observations About Being on Dialysis and Living With Chronic Kidney Disease

The second theme emerged surrounding observations about being on dialysis and living with chronic kidney disease. People wanted to be able to tell their story and share their experiences of living with chronic kidney disease and being on dialysis. The topics in this theme were not necessarily directly related to issues pertaining to challenges of getting hospital-based dialysis or the benefits and challenges of implementing peritoneal dialysis in their homes/communities; however, they did relate to their story of being a dialysis patient, including what led up to them having to go on dialysis in the first place.

#### Caregiver burden

Caregivers shared feelings of having to act like a parent and police their spouse’s eating and exercise. Frustration and strain on household duties was reported as the spouse was unable to contribute to household tasks like they used to.

#### Physical effects of being on dialysis

The physical toll dialysis has on patients includes exhaustion and lack of appetite. Many caregivers described episodes of patients being extremely weak and even passing out after treatment.

. . . and you go there and the next day you’re just feeling sick like he often was feeling sick the next day and then the second day he would be feeling better again and then it’s time to go back to the hospital so it just drains you right out and you’re sick . . .

#### Mental health effects of being on dialysis (and being diagnosed with chronic kidney disease)

Being diagnosed with chronic kidney disease or being on dialysis caused patients to experience depression and feelings of isolation. Patients identified the need to be in the proper mindset to make lasting changes to lifestyle.

#### Burden of traveling for hospital-based dialysis

The burden of traveling for hospital-based dialysis was large. Many issues were discussed around travel and included long distance, poor road conditions, weather, difficulty finding willing drivers, insufficient reimbursement for travel/meals/accommodations, the large time commitment spent traveling, and having dialysis take up their whole life.

It’s almost impossible to live in a community like this and do blood dialysis because it . . . you spend half your life in a taxi. Like, you go Monday, you come back Tuesday. You go Wednesday, you go back Thursday. So, three times a week. It’s almost your whole life. (On the challenges of not having peritoneal dialysis in the community.)

Dialysis all need an escort and escorts aren’t recognized as helpers. They’re just put there. Nothing is . . . because when patients get off dialysis, they’re awfully weak and tired and everything, and they all need an escort to help them at all times. And Health Canada does not recognize an escort. And medical drivers too that have been traumatized by what happens with the dialysis patients, a lot of things happen like passing out and stuff like that. Things we can’t deal with. Escorts have to deal with that and drivers have to deal with that. It’s not only being there for them. We help them along as much as we could cause I know, I’m always with him, and he’s passed out a few times on me where it was hard for me to revive him and I’m not a strong woman, but sometimes I have to lift him when he passes out on me, as an escort.

#### Benefits of home-based dialysis

Patients indicated the benefits of being on home-based dialysis as the ability to still work, an improved quality of life, and freedom with scheduling and not being tied to the dialysis machine.

He can hold a full time job all day then he just has to make sure he does his therapy at night. It’s like nine hours he’s connected to this machine.

#### Lifestyle that led to development/worsening of diabetes

Patients identified unhealthy food choices and lack of exercise as contributing to their chronic kidney disease. Solutions to address food security and healthy food choices were identified by one reserve and a pilot project was being implemented to try and give children a better chance at disease prevention.

. . . the community is going to come together and the community is going to get a voice and say how they’re going to try and bring local foods, like traditional foods into the schools and into the community as a whole . . . But whatever it is we’ll be really interested in to see if it’s more gardening or it’s learning to fish or hunt or getting wild meats into the schools but whatever happens it’ll be helpful for sure. And you know obviously the target age group is children but what a great place to start for prevention

#### Social determinants of health

Issues such as poor food security, poor housing conditions, and finances all were identified as contributors to developing chronic kidney disease. Some projects were being undertaken to address food security: community chest food bank and the aforementioned traditional foods research project.

#### Health care needs in the community

Participants in the sharing circles emphasized the importance of incorporating alternative/traditional medicine into the overall treatment plan for patients with chronic kidney disease. The importance of having regular access to a dietitian for prevention of diabetes but also for ongoing support to those with chronic kidney disease was highlighted.

## Discussion

The principal findings of this research were categorized under the main theme of underutilization of home dialysis and was further explored and documented under five subthemes including logistics, education and information, training and support, community support, and leadership and culture. Logistical issues were with equipment, infrastructure, supplies, and services. Areas identified as needing improvement to increase uptake of home-based dialysis among First Nations patients included appropriate education about chronic kidney disease and the overall safety of peritoneal dialysis, one-on-one training for patients requiring peritoneal dialysis, enhanced community support for patients and their caregivers, and delivery of services in a culturally supportive way. A secondary theme emerged as patients shared their stories of living with chronic kidney disease and being on dialysis. These topics included caregiver burden, the physical and mental health effects of being on dialysis, the burden of traveling, social determinants of health, and broader health care needs of the community.

The main strengths of this study were the inclusion of participants in a culturally meaningful way by utilizing sharing circles as the main source of information gathering as opposed to other methods which are not supported by First Nations such as survey type questionnaires. Another strength of this research was the robust working group and inclusion of many stakeholders including nephrologists, members from the Kidney Health Program, the Federation of Saskatchewan Indian Nations, Health Canada, and local community leaders. This robust working group ensured the research and data collection was done in a culturally sensitive way. Suggestions for improvement were innovative and bore out of the framework utilized by the working group. Potential limitations of this research include the possibility that the information gathered through the sharing circles and interviews is not represented of all First Nations in Saskatchewan. The sharing circles were held in geographically different communities to try and capture a representative sample of experiences and perceptions of patients, their families, health care providers, and community members and leaders. However, without actually engaging every community there could be barriers that have been missed. Participant characteristics (patient, caregiver, nurse, etc.) were not captured when speaking in the sharing circles, and therefore participants are not classified when quoted. Another limitation was the limited number of patients currently on home-based peritoneal dialysis and therefore their perceptions may not be adequately captured. Nevertheless, the information obtained is based on real-life local experiences as told by patients, family members, and service providers who live and work in the community.

Our findings echo previous work of some authors and contrast significantly with others. Previous research done by Salvalaggio et al documented the impact and experiences dialysis had on Aboriginal patients. They identified the following: physical symptoms, loss of independence, altered interactions with family and friends, and psychological adaptation to illness.^11^ The results of this trial provide some insight into important elements of the dialysis experience for patients and caregivers. Our participants echoed similar experiences with physical symptoms, isolation from the community, and the mental health component of being diagnosed with a chronic disease or having to go on dialysis. For future research, Salvalaggio et al suggested utilizing sharing circles as an ideal setting to explore the experience of dialysis from the perspective of patients and family and community members^11^ which is exactly what our research did.

A prospective cohort study by Mathew et al utilized self-reported patient surveys in patients with end-stage renal disease and found that Aboriginals reported lack of money and anxiety as significant barriers to the use of home-based peritoneal dialysis when compared with non-Aboriginal patients.^16^ In contrast to the self-reported surveys from Aboriginal patients, our methodology was quite different and may be the reason the barriers our research identified were different. Logistics such as housing, clean water, and reliable electricity may be similar in what Mathew et al reported as “lack of money” when trying to prepare a home for dialysis treatments.^16^ Anxiety was not found to be a barrier to starting home-based peritoneal dialysis but fear of negative outcomes and lack of support and health services were the main barriers identified. Patients did report that living with chronic kidney disease and being on dialysis had an impact on their physical and mental health but those impacts were not the reasons why home-based dialysis therapies were not implemented.

Another topic discussed through our research was a lack of support or physical limitations as barriers to initiating home-based peritoneal dialysis. The burden of home-based treatments for patients who had physical limitations like blindness to complete the treatments or require that level of assistance from family or community members on a regular basis was too much to ask for some patients. Previous literature has identified that Aboriginal patients have diabetes-caused end-stage kidney disease 84% of the time versus 37.3% of the time.^16^ It is plausible that the physical limitations caused by diabetes such as blindness or severe neuropathy are physical barriers to implementing home-based peritoneal dialysis more so than other conditions that require patients to go on dialysis.

Rix et al solicited Australian Aboriginal patients perspectives and experiences on hemodialysis with the goal of improved provision of services.^17^ Patients and their caregivers suggested home renal nurse support to enable patients to utilize home-based therapy and reduce the strain on families with having to travel and support a family member on hospital-based therapy. Home renal nurses were also suggested as a way to improve treatment compliance.^17^

Ongoing support was definitely a subtheme discussed in the sharing circles and was one of the reasons many individuals decided to seek dialysis treatments in the hospital as they felt more supported than if they were home alone. Suggestions for enhanced community services and supports were highlighted in our results and similar in the suggestions made by Rix et al.

Education and trust in information were identified as barriers to implementing home-based peritoneal dialysis. One patient had known 3 people to die early and all were on peritoneal dialysis. The literature is conflicting when it comes to Aboriginal mortality and the use of peritoneal dialysis. Sood et al determined that Aboriginals on peritoneal dialysis had higher mortality than Caucasians on peritoneal dialysis as well as higher peritoneal dialysis technique failure rates.^18^ However, Tonelli et al reported no significant difference in mortality between Aboriginal and non-Aboriginal peritoneal dialysis patients.^5^ If patients or members of the community have not observed positive outcomes for those who utilized home-based peritoneal dialysis, it is not surprising that mistrust in information or mistrust in the education provided by those supporting the implementation of home-based peritoneal dialysis emerged as a subtheme.

McLaughlin et al identified barriers to home-based dialysis through surveys completed by all patients with end-stage renal disease as (a) the belief that patients should not be dialyzed without direct supervision, (b) fear of social isolation, and (c) lack of space at home. The trial then further utilized educational interventions that targeted these frequently identified barriers and found that patients assigned to the education intervention group were significantly more likely to choose self-care dialysis.^19^ The subthemes of education and logistics were highlighted by participants in our research. Participants acknowledged that they were provided the option and information about both modes of dialysis; however, the delivery of the information or the validity of the information was not well-received. Another participant indicated the information that was provided was placating instead of being realistic about the severity and trajectory of the disease. Our research also identified lack of space at home for supplies as a barrier to utilizing home-based peritoneal dialysis as the preferred method of dialysis.

One implication of our research was the need for caregivers and policymakers to be aware of the cultural differences identified between First Nations and non-First Nations when it comes to advocating for their care. Culturally it was identified that it is not normal for a First Nations person to ask for help.

First Nations people are not apt to demand things while other cultures do demand things.

The requirement to ask for assistance from their community or family is not part of First Nations’ culture, making it difficult to implement a home-based peritoneal dialysis routine which does require ongoing support. Our research also highlighted the importance of health care providers not taking First Nations beliefs for granted and although certain values such as respect for dignity, noninterference, sharing, and the importance of family and community are widespread these need to be explored carefully with each person.^20^

Furthermore, highlighted was the need for First Nations communities to deliver educational and support services to patients in their home and community. Pamphlets and handouts are not a substitute for one-on-one patient education. Teaching circles and the use of translators may provide opportunities for patients to better understand their disease and also increase their confidence in choosing home dialysis.

The results of this research can be used as a support to address food and water security on reserves and further emphasize the need for reliable electricity. These results can be informative for policymakers and leaders when designing or working to improve diabetes and chronic kidney disease services for First Nations. The solutions suggested by participants were innovative and informative and underscore the importance of utilizing a grassroots approach when conducting future research.

## Conclusion

The processes and methods employed in this project served to engage patients, caregivers, community members, and health care providers in a culturally meaningful way. Although the process did seek to answer questions regarding home-based dialysis, it also opened up a collaborative engagement process on broader health services and outcomes.

Barriers to home-based peritoneal dialysis were identified as inadequate education and information, lack of community support, cultural disparities, intensity of training, and logistics. Suggestions for improvement had similar themes of community support, cultural awareness, and the importance of building trusting relationships. This research provides useful information about the barriers First Nations have faced in accessing home-based dialysis and the suggestions for improvement in the care of those with renal disease.
